# Previous SARS-CoV-2 Infection Status Among the Current RT-PCR-Positive Individuals Affected During the Second Wave of COVID-19 Infections in Chennai, India

**DOI:** 10.3389/fpubh.2022.836454

**Published:** 2022-04-04

**Authors:** Jeromie Wesley Vivian Thangaraj, Muthusamy Santhosh Kumar, C. P. Girish Kumar, Pragya Yadav, D. Sudha Rani, T. Karunakaran, Manoj Murhekar

**Affiliations:** ^1^ICMR-National Institute of Epidemiology, Chennai, India; ^2^ICMR-National Institute of Virology, Pune, India

**Keywords:** SARS-CoV-2, reinfection, IgG antibody, genomic sequencing, COVID-19

## Abstract

India witnessed a very strong second wave of coronavirus disease 2019 (COVID-19) during March and June 2021. Newly emerging variants of concern can escape immunity and cause reinfection. We tested newly diagnosed COVID-19 cases during the second wave in Chennai, India for the presence of Immunoglobulin G (IgG) antibodies to estimate the extent of re-infection. Of the 902 unvaccinated COVID-19 positive individuals, 53 (26.5%) were reactive for IgG antibodies and non-reactive for Immunogobulin M (IgM) antibodies. Among the 53 IgG-positive individuals, the interval between symptom onset (or last contact with the known case in case of asymptomatic) was <5 days in 29 individuals, ≥5 days in 11 individuals, while 13 asymptomatic individuals did not know their last contact with a positive case. The possible re-infections ranged between 3.2% (95% CI: 2.2–4.5%) and 4.3% (95% CI: 3.4–6.2%). The findings indicate that re-infection was not a major reason of the surge in cases during second wave. The IgG seropositivity among recently diagnosed unvaccinated COVID-19 patients could provide early indications about the extent of re-infections in the area.

## Introduction

After the first wave of COVID-19 in India, the transmission of wild Wuhan virus strain was relatively lower between October 2020 and February 2021 (1). The seroprevalence of Immunoglobulin G (IgG) antibodies against SARS-CoV-2 at the national level was around 25% at the end of December 2020 (2). India witnessed a very strong second wave of COVID-19 since March 2021 (3). The upsurge of COVID-19 cases seen in India in March 2021 was thought to be on account of several factors including high population susceptibility [75%, per national serosurvey in Dec 2020 (2)], non-adherence to non-pharmaceutical interventions, mass gatherings, and the emergence of variants of concern (VOC). Although there were limited data about predominantly circulating VOC from different Indian states before the second wave, the sequencing of >10,000 samples indicated circulation of viruses of B.1.1.7 (alpha variant), B.1.351 (beta), P.1 (Gamma) lineage, and delta (B.1.617) (4). Some VOCs can escape immunity and cause re-infection (5). A similar resurgence of COVID-19 occurred in Manaus, Brazil, despite high seroprevalence (6).

The definition of reinfection for COVID-19 has evolved over time. Most studies defined reinfection as infections with two distinct virus variants with any sequence variation between the two episodes (7). However, the confirmation of re-infection based on next-generation sequencing (NGS) is challenging, as specimens from the first infection are often not available. Moreover, in several countries, the facilities for genomic sequencing are limited. Some studies also used a time interval of at least 3 months between two real-time PCR (RT-PCR) positive tests as a criterion for defining reinfection (7). Most COVID-19 infections are mild or asymptomatic in nature, and several of such individuals with asymptomatic or mild infection would not undergo RT-PCR testing. During the upsurge of COVID-19 cases in Chennai, a metropolitan city in Southern India during March 2021, we attempted to quantify the proportion of reinfection among RT-PCR-confirmed COVID-19 cases, using serological signatures created by SARS-CoV-2 due to previous exposures. During October-November 2020, about 40% of the population aged >10 years had IgG antibodies against SARS-CoV-2 (8).

## Methods

As per the triaging protocol followed in Chennai, a line list of RT-PCR-positive individuals from the public and private laboratories in the city was sent to frontline health workers in the community. The frontline health workers identified the listed patients and referred them to the nearest screening centers, where the patients who are RT-PCR-positive were clinically evaluated by a physician. Based on the clinical, laboratory, and radiological findings, patients were triaged for home quarantine or hospital admission.

We conducted the study in two triaging centers in Chennai: one in the northern and another in the southern part of Chennai, which handled the maximum number of cases. All RT-PCR-positive individuals triaged at these centers during March 31, 2021 and April 13, 2021 were enrolled in the study. After obtaining written informed consent, we collected information about demographic details, symptoms of COVID-19, previous infection history, and vaccination details. A total of 3-ml of blood was collected from the patients and sera were tested for the presence of SARS-CoV-2 IgG antibodies against nucleocapsid (Abbott, USA), S1-RBD (Siemens, Germany), and IgM antibodies against S1-RBD (Abbott, USA). The individuals with the presence of IgG antibodies but negative for IgM antibodies, and whose date of onset of symptoms (for symptomatic cases) or last contact with COVID-19 case (for asymptomatic cases) was <5 days prior to blood sample collection were considered as possibly re-infected. We collected nasal and oro-pharyngeal (N/OP) swabs from such individuals for the NGS using the Illumina Miniseq (Illumina, USA) platform. We also collected swabs from those who reported laboratory-confirmed COVID-19 in the past and those who received COVID-19 vaccines at least 14 days prior to RT-PCR confirmation.

Viral Nucleic acid was extracted from the nasopharyngeal/oropharyngeal swabs specimens using a MagMAX™ Viral pathogen nucleic acid isolation kit (Thermo Fisher Scientific, USA). The extracted RNA was quantified using the Qubit® 2.0 Fluorometer (Invitrogen, USA) with a Qubit RNA High Sensitivity kit. The host ribosomal RNA (rRNA) depletion was carried out using the NEB-Next rRNA depletion kit (New England Biolabs, USA) and the extracted RNA was re-quantified. Quantified RNA was used to generate genomic libraries for sequencing. The quantified libraries were normalized and loaded on the Illumina machine for sequencing. The paired-end FASTQ files generated from the MiniSeq machine were analyzed on the CLC Genomics Workbench version 20 (CLC, Qiagen, Germany). A reference-based assembly method, as implemented in the Workbench, was used to retrieve the (Severe Acute respiratory Syndrome-2) SARS-CoV-2 sequence. The SARS-CoV-2 isolate Wuhan-HU-1 (Accession No.: NC_045512.2) was used as the reference for mapping. The retrieved sequences were deposited in the public repository, global initiative on sharing all influenza data (GISAID). Representative sequences were used in the analysis along with the sequences retrieved in this study. The aligned file was manually checked for correctness. A neighbor-joining (NJ) tree was constructed using the Tamura 3-parameter model, along with gamma distribution as the rate variation parameter. A bootstrap replication of 1,000 cycles was performed to assess the statistical robustness of the generated tree. The amino acid variation for each gene was identified using the MEGA software version 7.0 (9).

The study was approved by the Institutional Ethics Committee of ICMR-National Institute of Epidemiology, Chennai.

## Results

We enrolled 1,006 consecutive RT-PCR positive individuals between March 31 and April 13, 2021. Their mean age was 37.5 years (SD: 14), 808 (80.3%) were symptomatic, 5 had a history of COVID-19, and 104 reported receipt of the COVID-19 vaccine (Table 1).

**Table 1:** Demographic and clinical characteristics of the study participants.

**Characteristics**	**Number of study** **participants (% of the total)** ***N* = 1006**
**Age (in years)**	
10–18	65 (6.5)
19–45	661 (65.7)
46–60	208 (20.7)
61–85	72 (7.2)
Mean (SD)	37.5 (14.0)
**Gender**	
Male	625 (62.1)
Female	374 (37.2)
Transgender	7 (0.7)
**Reason for RT-PCR testing**	
Symptomatic	783 (77.8)
Contact with COVID-19 confirmed case	201 (20.0)
Medical Procedures	35 (3.5)
Travel	31 (3.1)
Others	88 (8.7)
**Presence of symptom at the time of enrolment**	
Asymptomatic	198 (19.7)
Symptomatic	808 (80.3)
**Symptoms (*****n*** **= 808)**	
Fever	574 (57.1)
Cough	268 (26.6)
Sore throat	190 (18.9)
New loss of smell	188 (18.7)
Excessive tiredness	176 (17.5)
New loss of taste	165 (16.4)
Diarrhea	64 (6.4)
Shortness of breath/difficulty in breathing	42 (4.2)
History of contact with COVID-19 case in the past	17 (1.7)
History of COVID-19 in the past among household member	65 (6.5)
History of previous COVID-19 infection	5 (0.5)
**COVID-19 vaccination status**	
One dose	94 (9.3)
Two doses	10 (1.0)
Unvaccinated	902 (89.6)

**Figure 1:**
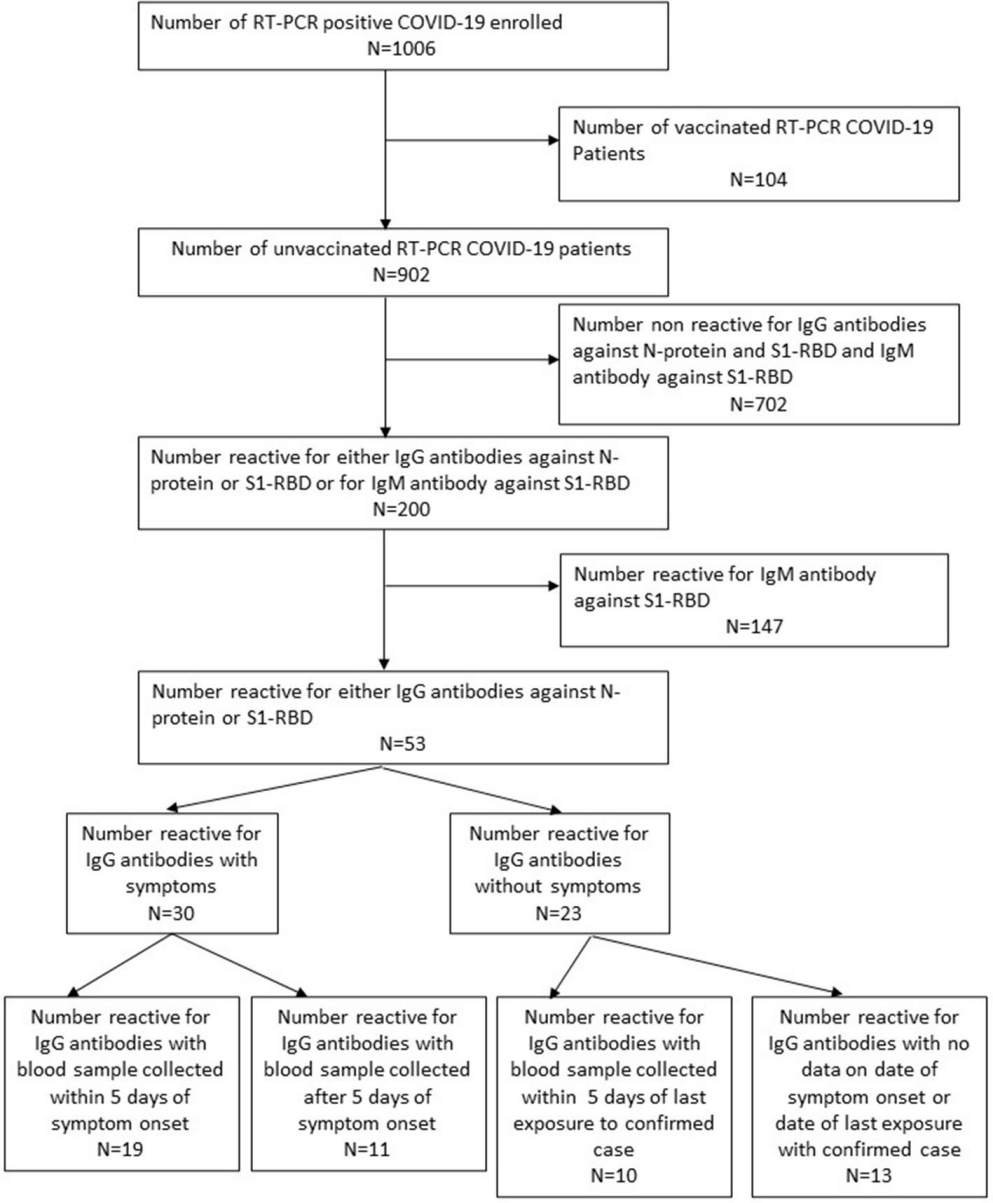
Flow chart describing the enrolment of study participants and their serological details.

Of the 902 unvaccinated RT-PCR-positive individuals, 702 (77.8%) were seronegative for IgG and IgM antibodies. A total of 147 (73.5%) of the remaining 200 were positive for IgM antibodies, whereas 53 (26.5%) were reactive for IgG antibodies (19 against nucleocapsid, 11 against S1-RBD, and 23 against both), and non-reactive for IgM antibodies. Three of the 5 individuals with a history of COVID-19 were reactive for IgG S1-RBD, while the remaining 2 were seronegative. Among the 53 IgG positive individuals, the interval between symptom onset (or last contact with a known case in case of asymptomatic) was <5 days in 29 individuals, ≥5 days in 11 individuals, while 13 asymptomatic individuals did not know their last contact with a positive case (Figure 1). Thus, the number of possible reinfections among the 902 RT-PCR-positive individuals ranged between 29 (3.2%, 95% CI: 2.2–4.5%) and 42 (4.3%, 95% CI: 3.4–6.2%) (assuming the interval between blood sample collection and last contact with the confirmed case was <5 days for all 13 asymptomatics).

**Figure 2:**
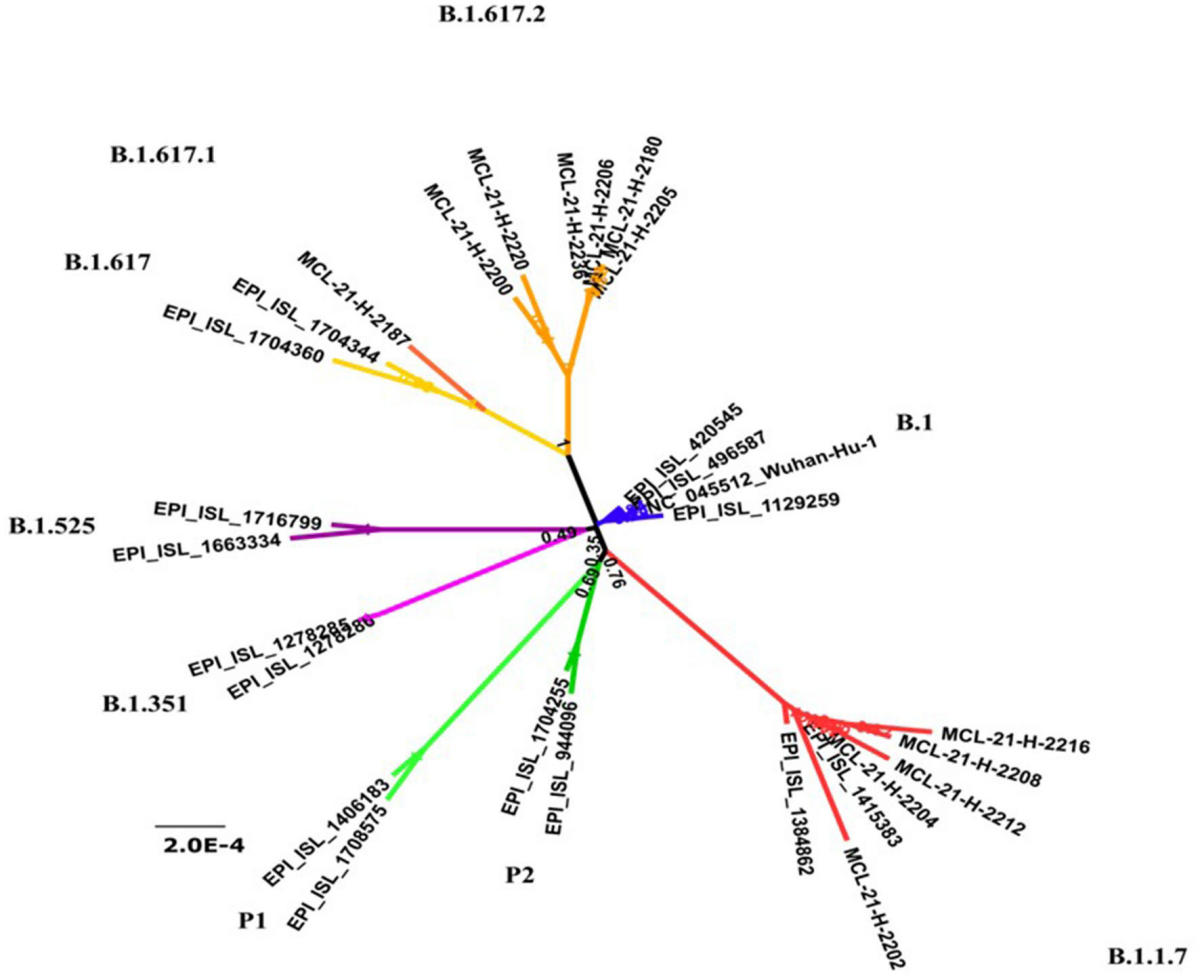
Phylogenetic trees of recovered 12 severe acute respiratory syndrom coronavirus 2 (SARS CoV-2) sequences and GISAID representative sequences to depict the presence of different lineages of the virus in Chennai.

We collected N/OP samples from 42 COVID-19 cases at the time of triage (29 with a history of vaccination and 2 with a history of COVID-19) or after serological testing (*n* = 11). We could retrieve 12 sequences; five belonged to B.1.1.7 (alpha variant) lineage, six belonged to B.1.617.2 (delta), and one B.1.617.1 (kappa) (Figure 2). Complete genome sequences could not be retrieved from 10 of 11 samples collected from possibly reinfected individuals and one belonged to B.1.617.2 lineage.

## Discussion

Our study findings indicate a low proportion (<5%) of isolated IgG antibodies among unvaccinated COVID-19 cases, suggesting re-infection was not a major reason for the surge of cases in Chennai during March-April 2021. Genomic analysis indicated alpha and delta variants as the predominant VOCs circulating in Chennai. The B1 has been a widely circulating strain in India, but alpha and delta VOCs were not reported in Chennai until November 2020 (10) and were possibly introduced subsequently. The surge was primarily driven by the Delta variant as indicated by the studies conducted in due course of time (11).

Since estimating reinfection through NGS had logistics issues, we attempted to use a serological approach to estimate the extent of reinfection at the population level. A systematic review on time to seroconversion post-infection indicates that in previously uninfected individuals, the mean or median time for IgG seroconversion was 12–15 days post-symptom onset, with wide variation ranging between four to 73 days. For IgM antibodies, the mean or median time to seroconversion ranged from four to 14 days post symptom onset (12). Hence, we operationally defined that the presence of IgG antibodies before 5 days of symptom onset could be due to the persistence of IgG antibodies on account of the previous infection. Using this approach, we estimated that <5% of the RT-PCR-positive unvaccinated individuals in Chennai were possible re-infections. The seroprevalence of IgG antibodies against SARS-CoV-2 in Chennai during October-November 2021 was around 40%. If reinfection was driving the surge, we expected a larger proportion of our study participants with isolated IgG antibodies on account of prior infection.

Our study has certain limitations. The IgG antibodies against SARS-CoV-2 wane over time. Also, some of the infected patients could develop IgG antibodies before 5 days of illness (13). Hence, we might have under-estimated the proportion of re-infections in our study. We could collect N/OP swabs from 38% of the possibly re-infected individuals and their NP swabs were collected after serological testing and not at the time of triage. Furthermore, using the serological approach to estimate the extent of reinfection in a community might not be a suitable strategy to adopt in highly vaccinated areas. Our study was a snapshot of a specific community in a specific period of time, where seroprevalence estimates were known. We tried this approach in March to April 2021, when vaccination rates were very low in the community.

In lower- and middle-income countries (LMICs), where facilities for NGS are limited, the anti SARS-CoV-2 IgG seropositivity among recently diagnosed unvaccinated COVID-19 patients could provide early indications about the extent of re-infections in the area.

## Data Availability Statement

The sequencing data presented in the study are deposited in GISAID. The GISAID ID of these sequences are mentioned below: MCL-21-H-2187EPI_ISL_2036281;MCL-21-H-2200EPI_ISL_2036282;MCL-21-H-2204EPI_ISL_2036283; MCL-21-H-2212EPI_ISL_2036284; MCL-21-H-2220EPI_ISL_2036285; MCL-21-H-2236EPI_ISL_8065277; MCL-21-H-2206EPI_ISL_8065278; MCL-21-H-2180EPI_ISL_8065284; MCL-21-H-2205EPI_ISL_8065280; MCL-21-H-2216EPI_ISL_8065281; MCL-21-H-2208EPI_ISL_8065282; MCL-21-H-2202EPI_ISL_8065283. The raw serological data supporting the conclusions of this article will be made available on request without undue reservation.

## Ethics Statement

The studies involving human participants were reviewed and approved by ICMR-National Institute Institutional Human Ethics Committee. Written informed consent to participate in this study was provided by the participants' legal guardian/next of kin.

## Author Contributions

MM and JT had full access to all the data in the study and takes responsibility for the integrity of the data and for the accuracy of the data analysis, and statistical analysis. JT, MK, and MM: concept and design. MM, JT, CK, and MK: drafting of the manuscript. CK and PY: laboratory processing and testing of samples. DR, TK, CK, JT, and MK: supervision. All authors acquisition, analysis, or interpretation of data, and critical revision of the manuscript for important intellectual content. All authors contributed to the article and approved the submitted version.

## Funding

The study was conducted using intramural funds of ICMR- National Institute of Epidemiology, Chennai.

## Conflict of Interest

The authors declare that the research was conducted in the absence of any commercial or financial relationships that could be construed as a potential conflict of interest.

## Publisher's Note

All claims expressed in this article are solely those of the authors and do not necessarily represent those of their affiliated organizations, or those of the publisher, the editors and the reviewers. Any product that may be evaluated in this article, or claim that may be made by its manufacturer, is not guaranteed or endorsed by the publisher.
